# Metagenomic Identification of Bacterioplankton Taxa and Pathways Involved in Microcystin Degradation in Lake Erie

**DOI:** 10.1371/journal.pone.0061890

**Published:** 2013-04-24

**Authors:** Xiaozhen Mou, Xinxin Lu, Jisha Jacob, Shulei Sun, Robert Heath

**Affiliations:** 1 Department of Biological Sciences, Kent State University, Kent, Ohio, United States of America; 2 The CAMERA Project, Center for Research on Biological Systems and California Institute of Telecommunication and Information Technology, University of California San Diego, San Diego, California, United States of America; Uppsala University, Sweden

## Abstract

Cyanobacterial harmful blooms (CyanoHABs) that produce microcystins are appearing in an increasing number of freshwater ecosystems worldwide, damaging quality of water for use by human and aquatic life. Heterotrophic bacteria assemblages are thought to be important in transforming and detoxifying microcystins in natural environments. However, little is known about their taxonomic composition or pathways involved in the process. To address this knowledge gap, we compared the metagenomes of Lake Erie free-living bacterioplankton assemblages in laboratory microcosms amended with microcystins relative to unamended controls. A diverse array of bacterial phyla were responsive to elevated supply of microcystins, including *Acidobacteria, Actinobacteria*, *Bacteroidetes*, *Planctomycetes*, *Proteobacteria* of the alpha, beta, gamma, delta and epsilon subdivisions and *Verrucomicrobia*. At more detailed taxonomic levels, *Methylophilales* (mainly in genus *Methylotenera*) and *Burkholderiales* (mainly in genera *Bordetella*, *Burkholderia*, *Cupriavidus*, *Polaromonas*, *Ralstonia, Polynucleobacter* and *Variovorax*) of *Betaproteobacteria* were suggested to be more important in microcystin degradation than *Sphingomonadales* of *Alphaproteobacteria*. The latter taxa were previously thought to be major microcystin degraders. Homologs to known microcystin-degrading genes (*mlr*) were not overrepresented in microcystin-amended metagenomes, indicating that Lake Erie bacterioplankton might employ alternative genes and/or pathways in microcystin degradation. Genes for xenobiotic metabolism were overrepresented in microcystin-amended microcosms, suggesting they are important in bacterial degradation of microcystin, a phenomenon that has been identified previously only in eukaryotic systems.

## Introduction

Freshwater lakes are ecologically important and a major source of drinking water; thus maintaining and improving water quality in lakes is critical. Cyanobacterial (blue-green algal) harmful blooms (CyanoHABs) threaten water quality and the frequency and extent of these blooms are increasing worldwide [1]. One important harmful effect of CyanoHABs is production of cyanotoxins, such as microcystins, which have strong hepatotoxicity that can severely damage mammalian liver cells. Microcystins (MCs) are produced by several bloom-forming cyanobacteria that are common in freshwater lakes, including *Microcystis*, *Anabaena*, *Planktonthrix* and *Nostoc* [2].

**Figure 1:**
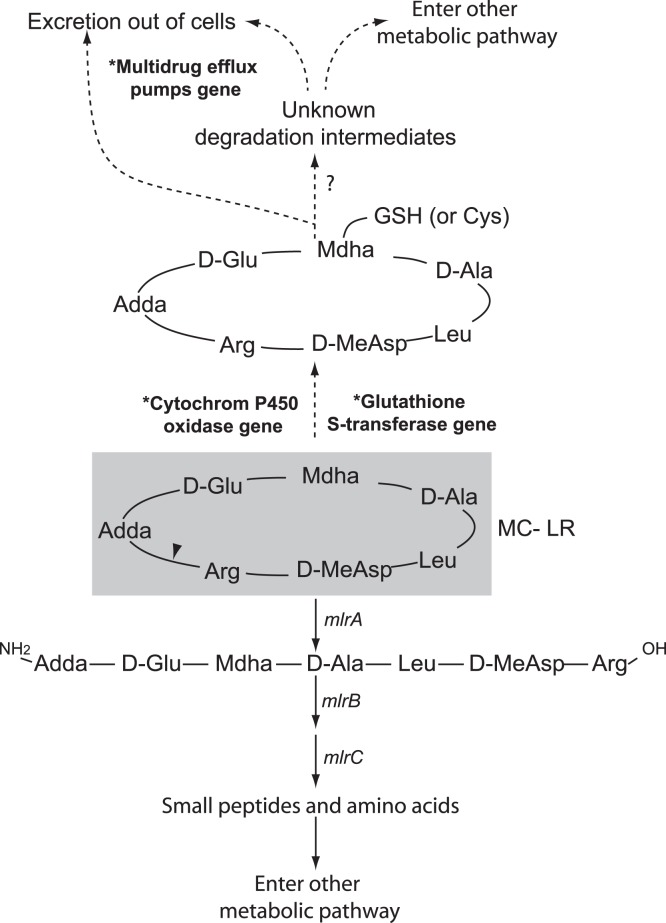
MC-LR structure and potential degradation pathways in bacteria. Steps of the known cleavage pathway are linked by solid arrows. Steps through xenobiotic metabolism are linked by dotted arrows. Overrepresented genes in the MC metagenomes are labeled with asterisks and bold fonts. Unknown genes are labeled with question marks. Triangle indicates the cleavage site of *mlrA*. Ala: alanine, Arg: arginine, Adda: 3-amino-9-methoxy-2, 6, 8-trimethyl-10-phenyldeca-4, 6-dienoic acid, Cys: cysteine, Glu: glutamic acid, GSH: glutathione, Leu: leucine, MeAsp: methylaspartic acid.

There are over 80 chemical variants of MCs, which all share a cyclic structure consisting of five constant non-protein amino acids and two variable protein amino acids [3]. Microcystin-LR (MC-LR) is the most abundant and well-studied form of MCs and contains leucine (Leu or L) and arginine (Arg or R) in the two variable positions (Figure 1). Due to its cyclic structure, MC-LR is chemically stable under the environmental range of pH, light radiation and temperature [4]. Heterotrophic bacterial assemblages are thought as major agents that regulate MC degradation in in lakes [5], [6], estuaries [7] and water treatment units [8].

Most previous studies on MC-degrading bacteria are culture-based and many of them are conducted in artificial environments, such as water treatment units [8], [9]. These studies have suggested that MC-degrading assemblages are mainly consisted of a narrow group of alphaproteobacteria in order *Sphingomonadales.* However, indirect evidence from studies on bacteria associated with MC-producing CyanoHABs has suggested that much broader bacterial taxa may be involved in MC degradation [10], [11]. Direct studies on *in situ* taxonomic composition of MC-degrading assemblages are scarce.

To date, a single pathway has been identified in bacterial systems for MC-LR degradation. This cleavage pathway is encoded by a cluster of genes (*mlrABCD*) and has been identified in all MC-degrading *Sphingomonas* species and several other strains of *Gammaproteobacteria* and non-*Sphingomonas Alphaproteobacteria* [3], [9], [12]. However, the specificity and ubiquity of *mlr* among environmental MC-degrading bacteria remain unclear [13]. This study aims to identify taxa, genes and pathways involved in microbially mediated MC transformation, using a comparative metagenomic approach on free-living bacterial assemblages from Lake Erie. Our results suggest that diverse taxa of free-living bacterioplankton, especially members of *Methylophilales* and *Burkholderiales,* might be important in MC degradation and that they likely employ different pathways from the *mlr*-based cleavage.

## Materials and Methods

### Sample Collection and Nutrient Analysis

Surface water samples were collected in carboys from the Western Basin of Lake Erie (Latitude 41.7423, Longitude −83.4019; Station MB18) on Aug. 27^th^, 2010, where CyanoHABs were reported throughout the summer, including at the time of this sampling trip (NOAA, Harmful Algal Bloom Events Response; http://www.glerl.noaa.gov). Before use, the carboys were acid washed in the lab and rinsed with ambient lake water three times. Standard limnological data were collected using a YSI 6600 Water Quality Sonde and included temperature, dissolved oxygen concentration, pH and turbidity (Table S1). The Secchi depth was also measured at the time of sampling. Water samples for nutrient analyses were filtered through 0.2 µm-pore-size membrane filters (Pall Life Sciences, Port Washington, NY) and stored on ice or at 4°C. Concentrations of nutrients, including dissolved organic carbon, total dissolved nitrogen, soluble reactive phosphorus, nitrate/nitrite, ammonium, were measured using standard methods for water quality analyses [14] and reported in Table S1.

### Microcosm Setup and Incubation

Lake water was filtered through 1.0 µm-pore-size membrane filters (Pall Life Sciences, Port Washington, NY) immediately after sampling to obtain free-living bacterioplankton proportion, and to exclude bacterivores and other large particles. Filtrate was collected in carboys and amended with a mixture of inorganic nitrogen and phosphorus compounds (5 µM NH_4_Cl, 5 µM NaNO_3_, and 1 µM NaH_2_PO_4_, final concentrations) and incubated in the dark at room temperature (22±1C°) with for 7 days. The water was agitated every 4–12 hours by shaking the carboys by hand. This pre-incubation was done to allow the bacteria to consume labile dissolved organic carbon compounds and to become growth limited by carbon availability.

At the end of the pre-incubation, microcosms were set up in six 20 L carboys. Two microcosms, designated as MC-1 and MC-2, were constructed of pre-incubated lake water and amended with MC-LR (∼15 µg L^−1^, final concentration, Axxora LLC, Farmingdale, NY). Two microcosms, designated as CT-1 and CT-2, served as controls and were constructed of pre-incubated lake water without further amendments. The remaining two microcosms were designated as FW-MC-1 and FW-MC-2; these received pre-incubated lake water that was in turn filtered by passage through 0.2 µm pore-size membrane filters to remove most of the bacterial cells and then amended with MC-LR (∼15 µg L^−1^, final concentration). The final volume of each microcosm was 18 L.

Microcosms were incubated in the dark at room temperature for a total of 48 hours and agitated every 4–12 hours by shaking the carboys by hand. Samples (10 ml) were taken in triplicates from each microcosm after 0 hour, 12 hours, 24 hours and 48 hours of incubation for subsequent MC-LR concentration measurement and flow cytometric analysis.

All plasticware was acid washed then rinsed with sample waters for three times before use. All glassware was ashed at 500°C for 5 hours then rinsed with sample waters for three times before use.

### Microcystin Concentration Measurement

Samples collected as above were filtered through 0.2-µm-pore size filters. MC-LR concentration in filtrate was measured using the Microcystins/Nodularins (ADDA) ELISA Kits (Abraxis BioScience, Warminster, PA) following the manufacturer’s instruction. Technical duplicates were measured for each sample.

### Flow Cytometric Analysis

Flow-cytometric analysis (FCM) was performed with a FACSAria (BD, Franklin Lakes, NJ) to measure the abundance, size and metabolic activity of bacterioplankton in the microcosms. Before FCM analysis, samples were preserved with 1% (final concentration) freshly made paraformaldehyde at room temperature for 2 hours. Preserved cells were stained with Sybr Green II (1∶5 000 dilution of the commercial stock; Molecular Probes Inc.) in the dark at room temperature for 20 min and mixed with an internal standard of beads that have a known density (1-µm-diameter Fluoresbrite YG Microspheres; Polysciences, Warrington, PA). FCM data acquisition was triggered by green fluorescence intensity of Sybr Green II staining (GFI). All FCM signals were collected on a logarithmic scale. Bacterial cell numbers were calculated based on ratios between the counts of bacterial cells and the internal bead standard.

FCM populations were defined based on GFI and side scatter (SSC) using a procedure described previously [15]. FCM population notation was based on the value of GFI from Sybr Green II staining, a proxy for intracellular nucleic acid content (largely RNA), which was taken as a surrogate indicator of cell activity [16]. Two FCM populations were gated for each sample, one was designated as “high intensity cells” (HI) and the other was designated as “low intensity cells” (LI). HI and LI populations thereby were corresponding to cells with higher and lower activity, respectively. Technical duplicates were analyzed for each sample.

### Bacterial Growth Rate Estimation

Bacterial growth rate (µ**)** during the incubation experiment was calculated using a linear regression formula: µ = (lnN_t_ –lnN_0_)/t, where t is the incubation time at the time of sampling, N_0_ and N_t_ are bacterial abundance at initial (0 hour) and at the time of sampling (t).

### DNA Extraction

For PCR amplification, bacteria cells in 1 L water samples were collected from each microcosm and filtered onto 47 mm-diameter, 0.2 µm-pore-size membrane filters (Pall Life Sciences, Port Washington, NY). Filters were changed after approximately every 500 ml of water filtered.

For metagenomic analysis, bacteria cells in ∼17 L water samples were collected from each microcosm and filtered on to 142 mm-diameter, 0.2 µm-pore-size membrane filters (Pall Life Sciences). Filters were changed after approximately every 9 L of water filtered. DNA was extracted from the filters using a PowerMax Soil DNA Isolation Kit (Mobio Inc, Carlsbad, CA) and served as templates for PCR amplification and metagenomic sequencing.

### 16S rRNA Gene Amplification and T-RFLP Analysis

16S rRNA gene amplification and terminal restriction fragment polymorphism (T-RFLP) analysis were performed following a protocol described previously with minor modifications [15]. Briefly, PCR was carried out with Illustra PuRe Taq Ready-to-go PCR beads (GE Healthcare, Piscataway, NJ) using 0.4 µM of 6-carboxyfluorescein (FAM) labeled 8F (5′-FAM-AGAGT TTGAT CCTGG CTCAG-3′) and unlabeled 1492R (5′-TACGG YTACC TTGTT ACGAC TT-3′) primers. A touchdown PCR program was used with the annealing temperature sequentially decreasing from 62°C to 52°C by 1°C per cycle, followed by 15 cycles at 52°C. Each PCR cycle included denaturing (at 95°C for 50s), annealing (at 62 to 52°C for 50s), and extension (at 72°C for 50s) steps. An initial 3-min denaturation and final 7-min extension step were also included. For each sample, triplicate PCR amplifications were performed and resulting amplicons were pooled before being examined on ethidium bromide-stained 1% agarose gels.

FAM-labeled PCR amplicons were purified with the QIAquick gel extraction kits (QIAGEN, Valencia, CA) and then digested with the CfoI restriction enzyme (Roche Applied Science, Indianapolis, IN) at 37°C for 3 hours. Afterwards, the digestion products were purified using ethanol precipitation. The length and relative abundance of each terminal restriction fragment (T-RF) were determined using a 3730 DNA Analyzer (Applied Biosystems) at the Plant-Microbe Genomic Facility, Ohio State University, Columbus, OH.

T-RFLP profiles among bacterial FCM populations were quantitatively compared using a hierarchical cluster analysis using the Primer v5 program (Primer-E Ltd, Plymouth, United Kingdom). The relative peak area of each terminal restriction fragment (T-RF) from the output of T-RFLP data was used as a proxy for the relative abundance of bacterial taxa associated with that T-RF peak. The relative peak areas were square-root transformed before analysis. T-RFs with <2% relative peak areas were excluded from the analysis.

### Metagenomic Sequencing and Sequence Annotation

Genomic DNA of metagenomes collected at the end of microcosm incubations, i.e., 48 hours after the MC-LR was added, was sequenced together by one full plate run of 454 multiplex pyrosequencing with titanium chemistry at the Georgia Genomics Facility, University of Georgia, Athens, GA. The metagenomic sequences were deposited in the CAMERA database under the project CAM_P_0000956.

Low quality reads (<200 bp or Phred quality scores <20) were removed from the metagenomic library. Identical reads that were generated as artifacts during pyrosequencing [17] were also removed using the CD-HIT-454 identifiers [18]. Remaining sequences were analyzed by BLASTn against the RDPII database to identify putative rRNA gene sequences (cutoff value of E <10^−5^). The taxonomic affiliations of each putative rRNA gene sequence was assigned based on the best hit of the BLASTn against the Greengenes database [19], using the E value <10^−10^ and identity >85%. The taxonomic annotation was further confirmed by consultation with RDP taxonomy classifier (>80% confidence).

Putative protein-coding sequences were identified from non-rRNA sequences by BLASTx against the NCBI RefSeq protein database (E ≤0.01, identity ≥40% and overlapping length ≥65 nt) [20]. The protein-encoding sequences were further categorized into Clusters of Orthologous Groups (COG) and Kyoto Encyclopedia of Genes and Genomes (KEGG) pathways by BLASTx against the NCBI’s COG database and the KEGG databases (E ≤0.1, similarity ≥40% and overlapping length ≥65 nt). The taxonomic affiliations were obtained by BLASTx against the NCBI RefSeq database using the MEGAN program [21]. Sequences that did not meet any of the criteria for rRNA or functional genes were excluded from further analysis.

### Functional Gene Identification

Putative *mlrABCD*, a cluster of genes that encode MC degradation in bacterial systems, were identified in the metagenomic libraries using tBLASTx with a bit score cutoff of 50. Putative glutathione S-transferase (GST) genes, key genes in xenobiotic metabolism, were identified using BLASTx with a bit score cutoff of 50. Accession numbers for reference gene sequences are provided in Table S2.

### Shannon-Wiener Index

The taxonomic diversity of microbes was estimated at the order level using the formula H’ = −∑ (P_i_ * ln P_i_), where P_i_ is the relative abundance of the sequences belonging to the ith microbial order, and R is the total number of unique orders.

### Statistical Analyses

A Student’s *t* test for two samples of unequal variance was performed to compare total bacterial abundance, relative abundance of each FCM population and MC-LR loss between the MC and CT microcosms.

A *t* test with Bonferroni correction for two samples of unequal variance [22] was used to compare the relative abundance of bacterial taxa at two levels, e.g., between the within-treatment metagenome replicates (MC1 vs. MC2 and CT1 vs. CT2) and between the pooled metagenomes of different treatments (MCs vs. CTs). Significant differences between MC and CT microcosms were reported at *P*<0.05 with Bonferroni correction. Taxa with significant within-treatment differences were removed from the final list of responsive taxa.

The Xipe-TOTEC program, a statistical method that has been specifically developed to compare metagenomes [23], was used to identify overrepresented COGs and KEGGs in the MC metagenomes. Pair-wise comparisons were performed between within-treatment metagenome replicates and between the pooled metagenomes of different treatments, based on the occurrence of gene categories. In each comparison, a total of 20,000 re-samplings were made, with the sample size equal to the average number of sequences in the two metagenome sequence libraries being compared. Significant differences between the metagenome datasets were reported at the level of *P*<0.02, after removing those gene categories that had significant within-treatment differences.

The results of Xipe-TOTEC are affected by sample size, i.e., the number of sequences in randomly formed pools [24], and the copy number of target genes [20]. Therefore, changes in relative abundance of gene categories were also assessed by a statistical analysis that is free of these concerns, which is based on calculating the odds ratio (OR) and binominal distribution probabilities [25]. OR was calculated using the equation [(n_mc_/(N_mc_-n_mc_)]/[n_ct_/(N_ct_-n_ct_)], where n_mc_ and n_ct_ were the number of targeted gene sequences in the pooled MC and CT metagenomes, respectively; N_mc_ and N_ct_ were the total number of sequences in the pooled MC and CT metagenomes, respectively. Binomial distribution of genes was assumed in each metagenomic sequence library. The binomial distribution probability (*P*) was calculated within Microsoft Excel, using the [n_mc_/(N_mc_-n_mc_)] as the observed gene sequence frequency and [n_ct_/(N_ct_-n_ct_)] as the expected gene sequence frequency. Genes or gene groups were reported as significantly overrepresented in MC metagenomes when the corresponding OR >1 and *P*<0.02. Genes or gene groups that had significant within-treatment differences were removed from the final list of overrepresented gene categories.

## Results

### Response of Bacterial Assemblages to Microcystin

**Figure 2:**
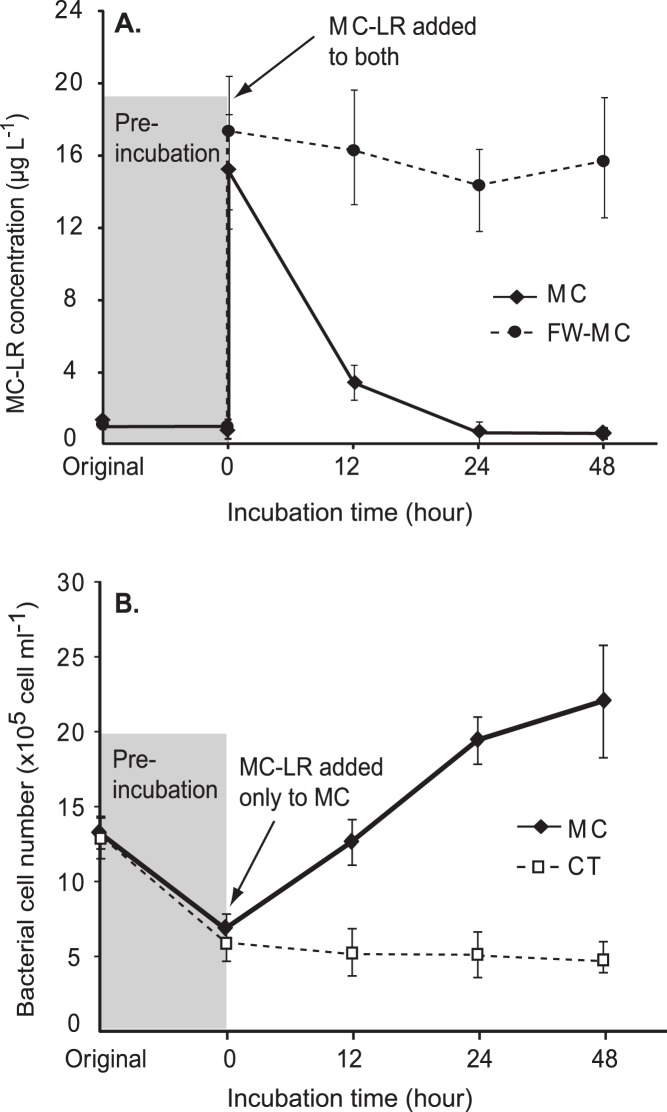
Variation of MC-LR concentration and total bacterial cell number during microcosm incubation. (A) Average MC-LR concentrations and standard deviations in the MC and FW-MC microcosms. (B) Average bacterial abundance and standard deviations in MC and CT microcosms. Shaded areas indicate periods of pre-incubation with inorganic N and P for establishing carbon-limited conditions in microcosms.

Added MC-LR was consumed rapidly in microcosms with a pre-established carbon-limited condition (Figure 2A). Within 12 hours of incubation, over 75% of MC-LR was lost and MC-LR became nearly undetectable after 24 hours in the MC microcosms. In contrast, MC-LR that was added to microcosms with filter-sterilized lake water (FW-MC) remained untransformed throughout the incubation (*t* test, *P*<0.05).

Concomitant with MC-LR consumption, number of bacteria in the MC microcosms significantly increased. Bacterioplankton in the MC microcosms nearly doubled within 12 hours following addition of MC-LR (growth rate; µ = 1.2 day ^−1^) and the cell density reached 2.2×10^6^ cells ml^−1^ after 48 hours of incubation (∼3.4-fold increase from initial cell density). Meanwhile, cell density in the control microcosms (CTs) was unchanged at 6.1×10^5^ cells ml^−1^ (*t* test, *P*<0.05; Figure 2B).

**Figure 3:**
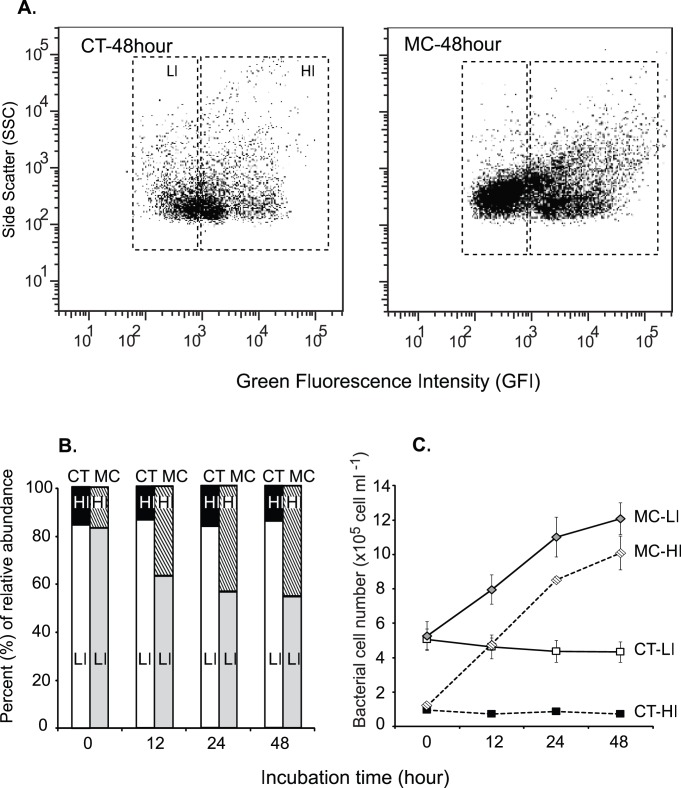
Distribution of bacterial cells of high (HI) and low (LI) metabolic activities during microcosm incubation. (A) Flow cytometric analysis of bacterial cell distribution in the MC and CT microcosms after 48 hours of incubation. (B) Average relative abundance of HI and LI cells in the MC and CT microcosms during the course of incubation. (C) Average numbers of HI and LI cells and standard deviations in the MC and CT microcosms during the course of incubation.

MC-LR addition also led to compositional differentiation between the MC and CT metagenomes (Figure 3). In the MC microcosms, the relative abundance of HIs increased from 14.3% of total cells (1.0×10^5^ cells ml^−1^) at 0 hour to 45.9% (3.1×10^5^ cells ml^−1^) at 48 hours of incubation. Meanwhile, no significant change was found for the relative abundance of HIs in the CT microcosms (*t* test, *P*<0.05; Figures 3B and 3C), indicating the observed increase in HI cells in the MC microcosms was due to growth of bacterioplankton on added MC-LR. At the end of the 48-hour incubation, LIs in the MC microcosms accounted for a smaller percentage (54.1%) than those in the CTs (85.7%), however, it contained nearly 3 times more cells than LIs in the CT microcosms (Figure 3C). This suggests that a considerable fraction of LI cells in the MC microcosms were also microcystin-responsive. Thus, rather than analyzing only the HI populations, the total bacterial communities (HI plus LI) in the MC and CT microcosms were examined to elucidate the taxa and genes involved in microcystin degradation.

**Figure 4:**
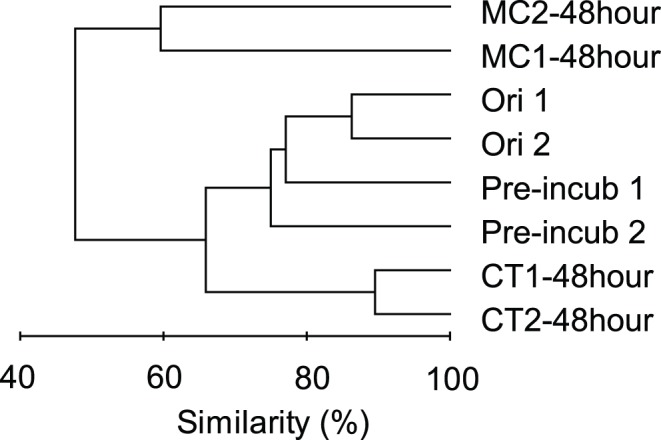
Clustering pattern of bacterioplankton 16S rRNA gene contents based on T-RFLP analysis. Cluster analysis of T-RFLP data for original water samples (Ori 1 and 2), samples at the end of inorganic nutrient pre-incubation (Pre-incub 1 and 2) and samples at the end of MC-LR incubation experiments in the microcystin amended (MC1–48 h and MC2–48 h) and control (CT1–48 h and CT2–48 h) microcosms.

T-RFLP analysis, based on 16S rRNA genes, was performed to examine the potential shifts in total bacterial community structures during inorganic nutrient pre-incubation and MC-LR incubation experiment. Cluster analysis of T-RFLP data closely grouped duplicates for each sample to each other, indicating good within-treatment reproducibility (Figure 4). The original water (Ori) and pre-incubated water samples (Pre-incub) had highly similar T-RFLP data. These two samples were moderately similar with the samples from the CT microcosms at the end of the incubation experiment (CT-48 h), but were distant from samples of the MC microcosms (MC-48 h) (Figure 4). These findings indicated that pre-incubation had less effect on bacterial community structure than MC-LR amendments.

### General Structure of Metagenomes

A total of 815,435 metagenomic sequences with average length of 386 bp were recovered, after removing low quality and artificial reads (Table 1). More sequences were recovered for the MC libraries than the CTs, although starting amounts of genomic DNAs were similar (∼1 µg). About 0.4% and 0.2% of the sequences of the MC and CT metagenomes, respectively, were affiliated with16S rRNA genes, in accordance with their expected frequency in prokaryotes (Mou *et al*., 2008). Most of the non-16S rRNA gene sequences in the MC (∼70%) and CT (∼50%) metagenomes were identified as putative protein-coding sequences.

**Table 1:** Sequence annotation statistics for the MC and CT metagenomes.

Parameter	MC-1	MC-2	CT-1	CT-2
Number of unique sequences	251,154	201,543	164,026	198,712
Average sequence length (bp)	386	366	414	377
Number (%) of total rRNA genes	1000 (0.4%)	771 (0.4%)	381 (0.2%)	359 (0.2%)
Number (%) of total predicted protein-coding genes	182,25 (73%)	140,86 (70%)	83,621 (51%)	91,778 (46%)
Number (%) of protein-coding genes categorized by COG groups	121,80 (67%)	92,719 (66%)	53,754 (64%)	61,742 (67%)
Number (%) of protein-coded genes categorized by KEGG pathways	168,118 (92%)	129,469 (92%)	72,542 (87%)	83,391 (91%)

**Figure 5:**
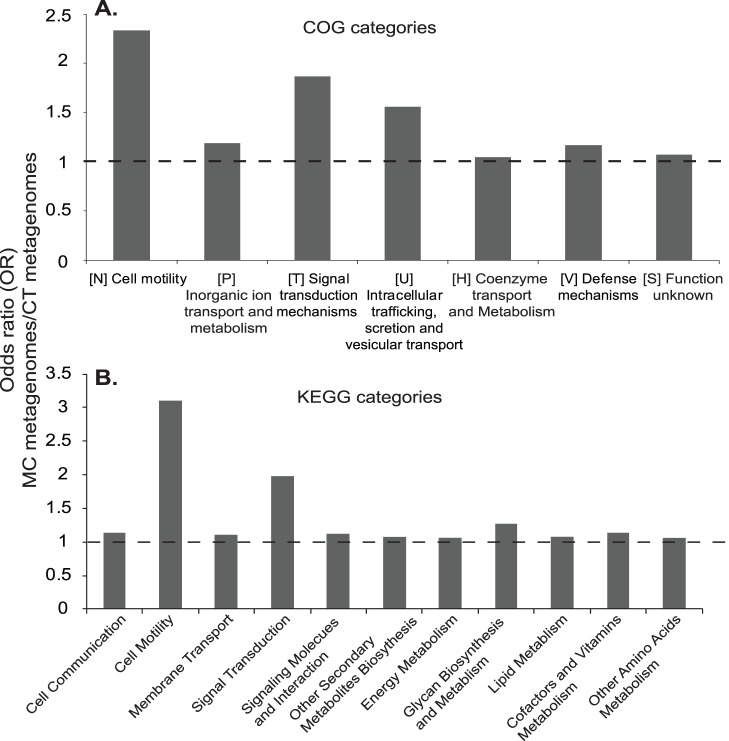
Significantly overrepresented gene categories in the MC metagenomes, relative to those in the CT metagenomes. (A) General COG categories. (B) General KEGG categories. Calculations were based on relative abundance of each gene categories between the MC and CT metagenomes. Significance overrepresentation was reported when OR>1, *P*<0.02.

Out of 498,519 putative protein-coding gene sequences, 90% were further assigned into a total of 3,259 unique COG groups and 182 unique KEGG pathways (Table 1). These were further classified into 23 COG (A-V, Z) and 21 KEGG classes within the networks of metabolism, genetic information processing, environmental information processing and cellular processes. COG and KEGG assignments consistently revealed that functional categories, including cell motility, signal transduction, metabolisms of organic and inorganic molecules were significantly overrepresented in the MC relative to the CT metagenomes (Figure 5).

### Microcystin Responsive COGs, KEGGs and Genes

Metagenomic contents of COG groups (COGs) and KEGG pathways (KEGGs) were compared between the MC and CT metagenomes using the Xipe-TOTEC (Rodriguez-Brito *et al*., 2006; *P*<0.02) and OR calculation analyses (Gill *et al*., 2006; OR >1, *P*<0.02). A total of 80 COGs and 14 KEGGs were found significantly overrepresented in the MC metagenomes relative to the CT metagenomes (Tables 2, 3,S3 and S5). These COGs and KEGGs were affiliated with metabolisms, mostly those for energy, amino acids, carbohydrates and lipids (28 COGs, 10 KEGGs), information processing (8 COGs, 2 KEGGs) and cellular processes, especially, bacterial motility and chemotaxis (33 COGs, 2 KEGGs; Figure 5). Several overrepresented COGs have been found in metagenomes associated with a microcystin-producing cyanobacterial bloom (Li *et al*., 2011), including COG0243 (anaerobic dehydrogenase), COG5013 (nitrate reductase alpha subunit), COG3696 (putative silver efflux pump), COG0583 (transcriptional regulator), COG0642 (signal transduction histidine kinase) and COG0664 (cAMP-binding proteins) (Table 2).

**Table 2:** Overrepresented COG groups in the MC metagenomes relative to the CT metagenomes, based on odds ratios (OR) calculated between the copy number of putative gene sequences in the MC and CT metagenomes.

COG^a^	COG description	Class	Class description	MC	CT	OR_MC/CT_
**Metabolism**						
0243*	Anaerobic dehydrogenases, typically selenocysteine-containing	C	Energy production and conversion	361	97	2.0
5013*	Nitrate reductase alpha subunit	C	Energy production and conversion	110	6	9.9
1362	Aspartyl aminopeptidase	E	Amino acid transport and metabolism	94	1	50.6
3696*	Putative silver efflux pump	P	Inorganic ion transport and metabolism	938	254	2.0
**Information storage and processing**						
0583*	Transcriptional regulator	K	Transcription	888	182	2.6
**Cellular processes and signaling**						
1291	Flagellar motor component	N	Cell motility	128	10	6.9
0643	Chemotaxis protein histidine kinase andrelated kinases	NT	Cell motility/Signal transduction mechanisms	348	64	2.9
0840	Methyl-accepting chemotaxis protein	NT	Cell motility/Signal transduction mechanisms	442	43	5.5
2804	Type II secretory pathway, ATPase PulE/Tfppilus assembly pathway, ATPase PilB	NU	Cell motility/Intracellular trafficking, secretion,and vesicular transport	551	166	1.9
3419	Tfp pilus assembly protein, tip-associatedadhesin PilY1	NU	Cell motility/Intracellular trafficking, secretion,and vesicular transport	113	11	5.5
5008	Tfp pilus assembly protein, ATPase PilU	NU	Cell motility/Intracellular trafficking, secretion,and vesicular transport	256	21	6.6
0625	Glutathione S-transferase	O	Posttranslational modification, protein turnover, chaperones	352	92	2.1
1391	Glutamine synthetase adenylyltransferase	OT	Posttranslational modification, protein turnover, chaperones/Signal transduction mechanisms	238	46	2.8
0642*	Signal transduction histidine kinase	T	Signal transduction mechanisms	962	343	1.5
0664*	cAMP-binding proteins - catabolite geneactivator and regulatory subunit ofcAMP-dependent protein kinases	T	Signal transduction mechanisms	367	95	2.1
0841	Cation/multidrug efflux pump	V	Defense mechanisms	1693	512	1.8
1566	Multidrug resistance efflux pump	V	Defense mechanisms	376	104	1.9

a Only those COG groups that were discussed in the present study or have been reported previously (labeled with asterisks) are shown. A full list is provided in Table S3.

**Table 3:** Significantly enriched KEGG pathways in the MC metagenomes relative to the CT metagenomes, based on odds ratios (OR) calculated between the copy number of putative gene sequences in the MC and CT metagenomes.

KEGG Pathway	General Processes	Functional Description	MC	CT	OR_MC/CT_
**Cellular Processes**					
2030	Cell Motility	Bacterial chemotaxis	2257	343	3.7
2040	Cell Motility	Flagella assembly	2458	518	2.7
**Environmental Information Processing**					
2020	Signal Transduction	Two-component system	8185	2297	2.1
3070	Membrane Transport	Bacterial secretion system	3063	1264	1.4
**Metabolism**					
0480	Metabolism of Other Amino Acids	Glutathione metabolism	2254	954	1.3
0540	Glycan Biosynthesis and Metabolism	Lipopolysaccharide biosynthesis	1822	539	1.9
0550	Glycan Biosynthesis and Metabolism	Peptidoglycan biosynthesis	2815	1279	1.2
0564	Lipid Metabolism	Glycerophospholipid metabolism	1542	613	1.4
0680	Energy Metabolism	Methane metabolism	3805	1807	1.2
0780	Metabolism of Cofactors and Vitamins	Biotin metabolism	469	136	1.9
0860	Metabolism of Cofactors and Vitamins	Porphyrin and chlorophyll metabolism	3160	1122	1.6
0910	Energy Metabolism	Nitrogen metabolism	2900	184	1.3
0920	Energy Metabolism	Sulfur metabolism	1379	535	1.4
0980	Xenobiotics Biodegradation	Xenobiotics metabolism by Cytochrom P450	342	124	1.5

Xenobiotic metabolism-related COGs and KEGGs were overrepresented in the MC metagenomes (Table 2). KEGG0980 and COG0625 are associated with cytochrome P450 oxidase and glutathione S-transferase (GST), respectively. These two enzymes have been found to catalyze the synthetic conversion of MC-LR into glutathione (GSH) and cysteine (Cys) conjugates in animal cells [26]. COG0841 and COG1566 are both affiliated with multidrug efflux pumps, which have been found to regulate the excretion of final degradation products of GSH and Cys conjugates from animal cells (Figure 1).

The known pathway of MC-LR cleavage in bacterial systems involves expression of a cluster of genes, e.g., *mlrABCD* [3], [9]. Putative *mlr* genes had similar relative abundance in the MC (0.22‰ of protein-coding sequences) and CT (0.19‰) metagenomes (OR >1, *P*<0.02). On the other hand, putative GST genes, which are involved in MC-LR degradation in animal cells but has yet unreported in bacteria [26], were overrepresented in the MC (0.54‰ of protein-coding sequences) than in the CT (0.24‰) metagenomes (OR >1, *P*<0.02).

### Microcystin Responsive Bacterial Taxa

Most of the putative protein sequences in the MC (90%) and CT (80%) metagenomes received taxonomic assignments at least to the phylum level, and ∼64% of these sequences had COG assignment. Patterns of taxonomic affiliation of metagenomic sequences were conserved, regardless of whether all protein-coding sequences or just those subsets assigned to significantly overrepresented COG categories were considered (Figure S1). In addition, even though 16S rRNA gene sequences were a much smaller fraction of total sequences than the protein-coding gene sequences (Table S4), they revealed a similar bacterial taxonomic structure (Figure S1 and Table S6).

**Figure 6:**
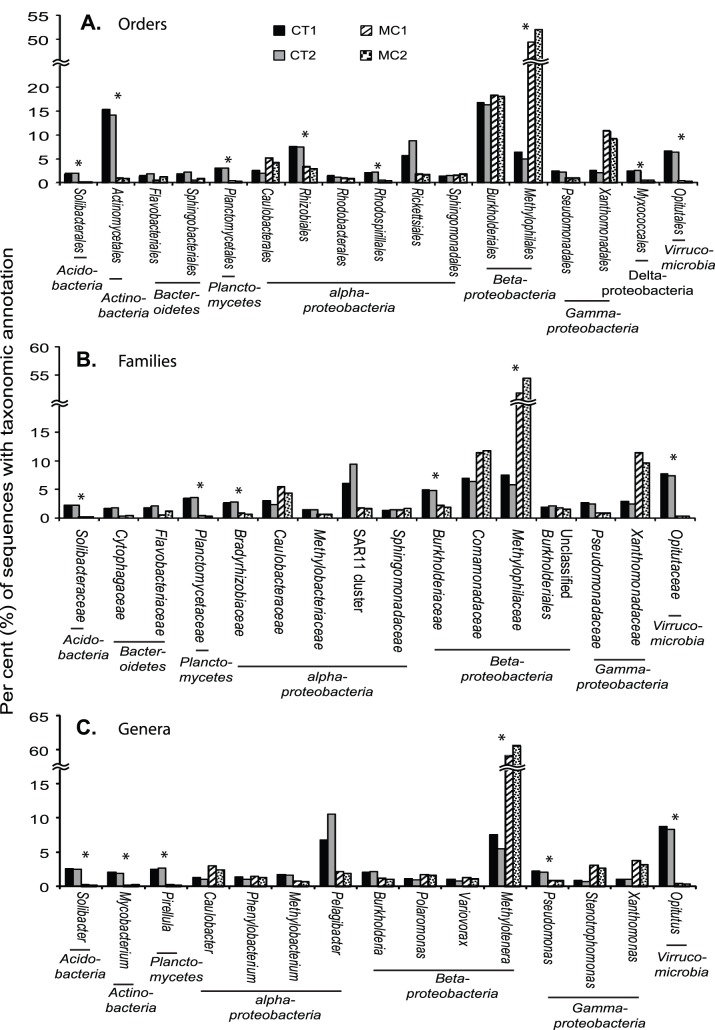
Taxonomic distribution of COG sequences in the MC and CT metagenomes. (A) At the order level. (B) At the family level. (C) At the genus level. Only major taxa are shown (collectively accounted for >4% of total metagenomic sequences). Asterisks are to label bacterial taxa with different relative abundance between the MC and CT metagenomes (OR >1, *P*<0.02).

COG sequences were affiliated with 89 unique bacterial orders, but about 65% of them were from only 22 orders of the phyla of *Acidobacteria, Actinobacteria*, *Bacteroidete*s, *Planctomycetes*, *Proteobacteria* (in subdivision of alpha, beta, gamma and delta/epsilon) and *Verrucomicrobia* (Figure 6A). Archaeal sequences occurred in low abundance (0.08% COGs in the MCs; 0.4% in the CTs) and 95% of them were affiliated with *Euryarchaeota*. The richness of the MC and CT metagenomes was similar at the order level. However, the COG sequences of the MC metagenomes were taxonomically less diverse (Shannon-Wiener Index, H’ = 2.0) than the CT metagenomes (H’ = 3.3), because evenness of the MC metagenomes was lower.

Over half (53.2%) of COG sequences in the MC metagenomes were affiliated with *Methylophilales* (*Betaproteobacteria*), a taxon that was significantly less abundant in the CT metagenomes (9.6% of COG sequence; *t* test with Bonferroni correction, *P*<0.05). *Burkholderiales* (*Betaproteobacteria;* 18.1% of COG sequences) and *Xanthomonadales* (*Gammaproteobacteria*; 9.0%) were the second and third most abundant taxa affiliated with COG sequences in the MC metagenomes. Their relative abundances were similar to those in the CT metagenomes (15.9% and 2.2%, respectively) (Figure 6).

Although representing fewer sequences (Table S4), similar distribution patterns of bacterial taxa were observed at the family and genus levels (Figures 6B and 6C). Like their parent order *Methylophilales*, the family *Methylophilaceae* and genus *Methylotenera* were the most abundant members in the MC metagenomes and were significantly more abundant than those in the CT metagenomes (*t* test with Bonferroni correction, *P*<0.05). On the other hand, underrepresentation of *Actinobacteria* in the MC metagenomes at the order level was not observed at the family or species level (Figure 6). This may be partly due to the fact that only a limited number of environmental *Actinobacteria* species have been isolated and sequenced [27].

**Figure 7:**
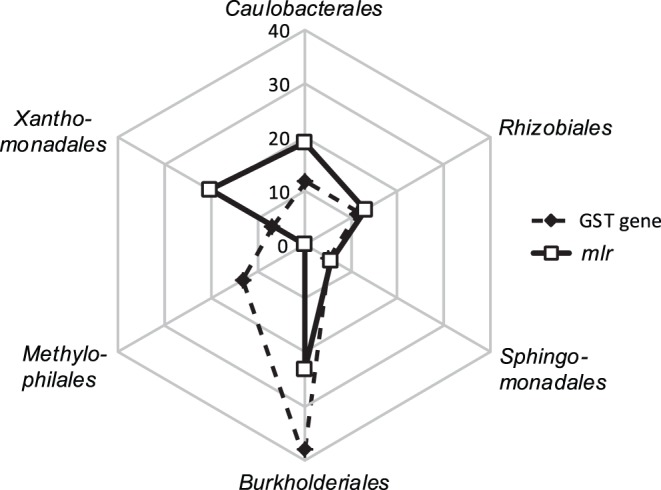
Percent distribution of major bacterial orders that were affiliated with GST and ***mlr*** **genes.**

Putative genes of MC-LR cleavage pathway (*mlr*) and xenobiotic metabolisms (GST genes) were affiliated with 13 and 16 bacterial orders, respectively (Figure 7). About 80% of the putative *mlr* sequences were affiliated with only 5 orders, including *Burkholderiales* (in genera *Burkholderia*, *Cupriavidus,* and *Variovorax*), *Caulobacterales* (*Phenylobacterium*), *Rhizobiales* (*Mesorhizobium*, *Methylobacterium* and *Rhodopseudomonas*), *Sphingomonadales* (*Sphingopyxis*), and *Xanthomonadales* (*Stenotrophomonas).* These orders and genera also represented major taxa for putative GST gene sequences. Taxonomic affiliations of *mlr* and GST genes were statistically similar between the MC and CT metagenomes (OR >1, *P*<0.02). However, a significant difference was found for *Methylophilales*-affiliated sequences. They accounted for over 13% of putative GST gene sequences but were not identified among the putative *mlr* sequences (Figure 7).

## Discussion

Bacterially mediated microcystin degradation has been studied primarily on bacterial cultures or in artificial environments. Related studies in natural environments have generally assumed that bacteria associated with CyanoHABs are predominant microcystin degraders [25], [26], [27], [28]. Using microcosm incubations, our study provides empirical data to identify bacterial genes and taxa that are involved in microcystin degradation in nature.

Microcosms are widely used in ecological research because they can be readily replicated and examined under controlled laboratory conditions, permitting experimental manipulations as in this study. However, the reliability of conclusions drawn from microcosms can be compromised by artifacts of confinement (“bottle effects”), which are exacerbated as the ratio of bottle surface to microcosm volume increases [29]. For this reason we constructed microcosms as large as possible to be manipulated in the laboratory: 18 L microcosms in 20 L carboys. Because of the uniformly large size of our microcosms we assumed that “bottle effects” would be consistent among the treatments and have low impacts on our overall conclusion. Other manipulations, i.e., pre-filtration and pre-incubation, were found necessary to establish contrasting results of cell abundance, size and nucleic acid content distributions, and MC-LR degradation activities between the MC and CT microcosms. However, these processing steps also made the experimental systems less *in situ*-like. Nonetheless, our approach allowed culture-independent identification of MC-degrading bacterial taxa and genes without constrains from prior knowledge.

In this study, free-living bacterioplankton grew substantially at the expense of added MC-LR, indicating they were actively using microcystin as carbon and/or energy sources (Figure 2). Cell distribution observed from FACS analysis also indicated that MC-LR affected the bacterial taxa differently and stimulated the growth of a subset of bacterial taxa present in the samples (Figure 3). The average growth rate of bacterioplankton in MC microcosms was 0.94 day^−1^, which is comparable to rates that have been reported for Lake Erie bacteria [30], [31], [32]. The consumption rate of MC-LR (∼15 µg L^−1^ in 48 hours) in microcosms was similar to previous observations in pure cultures growing on MC-LR at similar concentrations [8], [33]. If MC-LR were supplied at higher concentrations (>25 µg L^−1^) and/or to un-manipulated ambient lake water, which contains diverse labile dissolved organic compounds, MC-LR degradation rate would likely be slower and with a lengthy initial lag phase [8], [33].

Base on the amount of MC-LR addition and the growth of bacterial cells, we estimated that the average carbon content per bacterial cell (C_c_) in the MC microcosms was 6 fg/cell. The widely used average C_c_ for bacteria is 20 fg/cell [34], but many studies have proposed lower values (7–13 fg/cell) [35], [36], [37]. Studies have shown that C_c_ values of bacterial cells can range across three orders of magnitude, from1.5 fg/cell to 1.9 pg/cell [36]. Lower C_c_ values are typically associated with cells that are small in size [34], [36] and/or growing under nutrient limited conditions [38], similar as those in the MC microcosms. This calculation also indicated that bacterial growth in the MC microcosms can be largely explained by active incorporation of added MC-LR by bacterial cells.

Over twenty strains of MC-degrading bacterial isolates are currently available and they are affiliated with a narrow group of bacterial orders, including *Actinomycetales*, *Bacillales, Sphingomonadales* and *Burkholderiales* and *Methylophilales* [13], [39], [40]. Our culture-independent study suggests a highly heterogeneous composition of MC-responsive bacteria, including members from over 89 orders with in the phyla of *Actinobacteria*, *Bacteroidetes*, *Firmicutes*, *Planctomycetes*, *Proteobacteria* of the alpha, beta, gamma and delta/epsilon subdivisions, and *Verrucomicrobia*. Recent studies on CyanoHAB-associated bacteria have similarly indicated a high taxonomic diversity of MC degraders in a number of freshwater lakes [10], [41], [42].

Previous culturing studies and surveys of CyanoHAB-associated bacteria have suggested a dominant role of *Sphingomonadales* (mainly within the genus *Sphingomonas*) in MC degradation. However, a recent survey by 16S rRNA gene pyrotag sequencing has indicated a low relative abundance of *Sphingomonadales* (∼1% of total bacterial community) during a CyanoHAB event in Lake Erie [43]. Our metagenomic data also indicate that *Sphingomonadales* may be less important than *Methylophilales* and *Burkholderiales* in bacterioplankton-mediated MC degradation in Lake Erie (Figures 6 and S1; Table S6). The latter two *Betaproteobacterial* orders are common to freshwater environments [44], and each has cultured MC-degrading representatives [7], [40]. Moreover, although often at low abundance, *Methylophilales* have been frequently found to be associated with freshwater CyanoHABs [25], [26], [27], [28], [41]. Differences between our study and those of others most likely are due to variation between one site and another in physical, chemical and biotic conditions and the targeted fraction of the bacterioplankton (free-living bacterioplankton vs. total community). Notwithstanding those differences, our findings emphasize that MC-degrading bacteria and pathways likely are broader than earlier studies indicated.

Archaea have been identified as important *in situ* microbial taxa during a MC-producing CyanoHAB, but their high abundance has declined to undetectable levels after being incubated in MC-amended microcosms [10]. The authors have attributed this to high sensitivity of Archaeal cells to “bottle effects”. In our study, MC and CT metagenomes were subjected to the same container incubation conditions. Archaea-affiliated sequences occurred in low quantities in all microcosms, and their relative abundance was significantly lower in the MC (0.08% of total protein-coding sequences) than in the CT microcosms (0.5%) (*t* test with Bonferroni correction, *P*<0.05). This suggests an insignificant role of archaea in microcystin degradation.

*Actinobacteria* have several MC-degrading species and are common taxa associated with MC-producing CyanoHABs [10], [26]. They have been found more important during CyanoHABs in lakes with water temperature below 20°C than in warmer lakes. In our study, water temperatures at time of sampling and during incubation were at 22°C or above. Significantly fewer *Actinobacteria* were found in MC metagenomes, indicating that this taxon was insignificant in MC degradation in the samples examined.

The genes (*mlrABCD*), intermediates and products of an enzymatic pathway for bacterial MC degradation have been identified based on works on *Sphingomonadales* strains [12], [45]. *mlrA* genes are considered as the most important within the *mlr* cluster because they encode the ring-cleavage step that leads to opening of the microcystin ring structure (Figure 1). Probes/primers of *mlrA* have been developed and used to study *in situ* activity of MC-degrading bacteria in various environments [46], [47], [48]. However, PCR amplification of *mlrA* genes from microcystin-degrading *Actinobacteria* isolates has failed [13]. Two factors may be contributing to this failure: first, existing primers may be inefficient for broad identification of *mlrA* in non-*Sphingomonas* taxa [40] and second, microcystin degradation genes and/or pathways may vary among bacterial taxa. Our results support the latter hypothesis. In this study, putative *mlrA* genes were identified based on full-length amino acid sequence homology, which should have largely bypassed the bias of primer specificity that is inherent in the PCR method. In accordance, recovered *mlrA* genes in our metagenomes were broadly affiliated with *Proteobacteria* (in subdivisions of alpha, beta, gamma and delta) and *Bacteroidetes*. In addition, none of the *mlrA* or other *mlr* sequences was affiliated with *Methylophilales,* even though *Methylophilales* represented the most abundant taxon in the MC metagenomes. These suggest that bacteria, especially members of *Methylophilales*, may employ an alternative microcystin degradation pathway.

Our results suggest that this alternative pathway may involve xenobiotic metabolism (Figure 1). Xenobiotic metabolism-related genes and gene categories, e.g., GST gene and COG0625, COG0841, COG1566 and KEGG0980, were significantly overrepresented in the MC metagenomes. Moreover, *Methylophilales* were affiliated with a large proportion of xenobiotic metabolism related sequences, but none of the putative *mlr* sequences. Xenobiotic metabolism is widely distributed among living organisms of all three life domains and refers to intracellular processes that neutralize and eliminate toxic effects of foreign compounds by altering their chemical structures. A long list of substrates has been identified for xenobiotic metabolizing systems in bacteria, including halogenated compounds [49], drugs [50] and numerous environmental pollutants [51]. Our metagenomic study suggests adding MC-LR to this list. Although it is novel for bacterial systems, GST-mediated xenobiotic metabolisms are known for their critical role in MC detoxification by various aquatic eukaryotes, including higher plants, invertebrate and vertebrate animals [26]. The wide distribution of GST genes and related xenobitc metabolism have been largely attributed to horizontal gene transfer, and the parallel and independent evolution of these essential genes among different phylogenic groups [52].

It is noted that our results did not rule out the involvement of *mlr* gene-based pathway in MC-LR degradation, but suggested that alternative pathway, such as xenobiotic metabolism, may also be important in the process. Further studies, especially those that identify degradation intermediates and measure gene expression, are required to confirm the occurrence of MC degradation by xenobiotic metabolism in bacteria and to examine its importance relative to *mlr* gene-based cleavage pathway.

## Supporting Information

Figure S1 **Relative abundance of major bacterial taxa at order levels in the MC and CT metagenomes.** Taxonomic affiliations are based on (A) total protein sequences, (B) total protein sequences with COG group assignment, (C) COG sequences that were overrepresented in the MC metagenomes, relative to the CT metagenomes and (D) putative 16S rRNA genes.
(EPS)

Table S1 **Basic physiochemical parameters of surface water samples at the time of collection.**
(DOC)

Table S2 **NCBI database accession numbers for reference sequences used to identify homologs to 16S rRNA and functional genes.**
(DOC)

Table S3 **Full list of overrepresented COG groups in the MC metagenomes relative to the CT metagenomes.**
(DOC)

Table S4 **Number and percent of metagenomic sequences with taxonomic assignment at different resolution levels.**
(DOC)

Table S5 **List of underrepresented COG groups in the MC metagenomes relative to the CT metagenomes.**
(DOC)

Table S6 **Relative abundance (% of total sequences) of bacterial taxa revealed by 16S rRNA gene sequences in each metagenomes.**
(DOC)
