# Supplementary material for: Metagenomic Identification of Bacterioplankton Taxa and Pathways Involved in Microcystin Degradation in Lake Erie
Source: PLoS One. 2013 Apr 24;8(4):e61890. doi: 10.1371/journal.pone.0061890 (PMC3634838; doi:10.1371/journal.pone.0061890)
Supplement: Table S1 — Basic physiochemical parameters of surface water samples at the time of collection. (DOC) [file pone.0061890.s002.doc]

Table S1. Basic physiochemical parameters of surface water samples at the time of collection.

| **Parameter** | **Measurement** |
| --- | --- |
| Sampling depth | 0.345 m |
| Temperature (T) | 23.37 °C |
| Dissolved oxygen (DO) | 9.06 mg/L |
| DO saturation | 106.5% |
| pH | 8.57 |
| Turbidity | 14.9 NTU |
| Chlorophyll a | 3.6 μg/L |
| Secchi depth | 0.6 m |
| DOC concentration | 6.5 ± 0.1 mg/L |
| DON | 0.60 ± 0.04 mg/L |
| SRP | 12.6 ± 0.1 μg P/L |
| Nitrate/nitrite | 0.30 ± 0.1 mg N/L |
| Ammonium | 279.5 ± 15.6 μg N/L |
