# Supplementary material for: Metagenomic Identification of Bacterioplankton Taxa and Pathways Involved in Microcystin Degradation in Lake Erie
Source: PLoS One. 2013 Apr 24;8(4):e61890. doi: 10.1371/journal.pone.0061890 (PMC3634838; doi:10.1371/journal.pone.0061890)
Supplement: Table S2 — NCBI database accession numbers for reference sequences used to identify homologs to 16S rRNA and functional genes. (DOC) [file pone.0061890.s003.doc]

Table S2. NCBI database accession numbers for reference sequences used to identify homologs to 16S rRNA and functional genes.

| **Genes** | **Bacterial taxa** | **NCBI sequence accession number** | **References** |
| --- | --- | --- | --- |
| 16S rRNA gene | *Sphigomonas* sp. ACM-3962 | AF411072 | [1] |
| GST gene | *Sphigomonas wittichii* RW1 | YP_001264142; YP_001263941; YP_001260528; YP_001260623; YP_001264103; YP_001263178; YP_001264599; YP_001261939; YP_001260743; YP_001261352; YP_001265197; YP_001262742; YP_001264641; YP_001261016; YP_001263066; YP_001263373; YP_001264728; YP_001260654 | [2] |
| *mlrA*/*mlrD* | *Sphigomonas* sp. ACM-3962 | DQ423535 | [3] |
|  | *Sphingopyxis sp. LH21* | DQ423536 | [3] |
| *mlrB* | *Sphigomonas* sp. ACM-3962 | AF411069 | [1] |
| *mlrC* | *Sphigomonas* sp. ACM-3962 | AF411070 | [1] |
| *mlrC*/*mlrA-like* | *Sphigomonas* sp. ACM-3962 | DQ423533 | [3] |
|  | *Sphingopyxis sp. LH21* | DQ423534 | [3] |
| *mlrD* | *Sphigomonas* sp. ACM-3962 | AF411071 | [1] |

[1] Bourne DG, Riddles P, Jones GJ, Smith W, Blakeley RL. (2001) Characterisation of a gene cluster involved in bacterial degradation of the cyanobacterial toxin microcystin-LR. Environ Toxicol 16: 523-534.

[2] Miller TR, Delcher AL, Salzberg, SL, Saunders E, Detter JC, Halden RU. (2010) Genome Sequence of the Dioxin-Mineralizing Bacterium *Sphingomonas wittichii* RW1. J Bacteriol 192:6101-6102.

[3] Ho L, Hoefel D, Saint CP, Newcombe G. (2007a) Isolation and identification of a novel microcystin-degrading bacterium from a biological sand filter. Water Res 41: 4685-4695.
