# Supplementary material for: Metagenomic Identification of Bacterioplankton Taxa and Pathways Involved in Microcystin Degradation in Lake Erie
Source: PLoS One. 2013 Apr 24;8(4):e61890. doi: 10.1371/journal.pone.0061890 (PMC3634838; doi:10.1371/journal.pone.0061890)
Supplement: Table S3 — Full list of overrepresented COG groups in the MC metagenomes relative to the CT metagenomes. (DOC) [file pone.0061890.s004.doc]

Table S3. Full list of overrepresented COG groups in the MC metagenomes relative to the CT metagenomes^a^.

| **COG** | **COG description** | **Class** | **Class description** | **CT** | **MC** | **OR_MC/CT_** |
| --- | --- | --- | --- | --- | --- | --- |
| **Metabolism** | | | | | | |
| 0243 | Anaerobic dehydrogenases, typically selenocysteine-containing | C | Energy production and conversion | 97 | 361 | 2.0 |
| 0348 | Polyferredoxin | C | Energy production and conversion | 18 | 220 | 6.6 |
| 1018 | Flavodoxin reductases (ferredoxin-NADPH reductases) family 1 | C | Energy production and conversion | 40 | 203 | 2.8 |
| 1049 | Aconitase B | C | Energy production and conversion | 22 | 236 | 5.8 |
| 1229 | Formylmethanofuran dehydrogenase subunit A | C | Energy production and conversion | 6 | 80 | 7.2 |
| 1251 | NAD(P)H-nitrite reductase | C | Energy production and conversion | 30 | 214 | 3.9 |
| 4658 | Predicted NADH:ubiquinone oxidoreductase, subunit RnfD | C | Energy production and conversion | 2 | 72 | 19.5 |
| 5013 | Nitrate reductase alpha subunit | C | Energy production and conversion | 6 | 110 | 10.0 |
| 1362 | Aspartyl aminopeptidase | E | Amino acid transport and metabolism | 1 | 94 | 51.0 |
| 0418 | Dihydroorotase | F | Nucleotide transport and metabolism | 13 | 118 | 4.9 |
| 0120 | Ribose 5-phosphate isomerase | G | Carbohydrate transport and metabolism | 13 | 125 | 5.2 |
| 2956 | Predicted N-acetylglucosaminyl transferase | G | Carbohydrate transport and metabolism | 18 | 121 | 3.7 |
| 3957 | Phosphoketolase | G | Carbohydrate transport and metabolism | 16 | 151 | 5.1 |
| 5309 | Exo-beta-1,3-glucanase | G | Carbohydrate transport and metabolism | 3 | 87 | 15.7 |
| 0635 | Coproporphyrinogen III oxidase and related Fe-S oxidoreductases | H | Coenzyme transport and metabolism | 71 | 295 | 2.3 |
| 0654 | 2-polyprenyl-6-methoxyphenol hydroxylase and related FAD-dependent oxidoreductases | HC | Multiple classes | 111 | 414 | 2.0 |
| 1502 | Phosphatidylserine/phosphatidylglycerophosphate/cardiolipin synthases and related enzymes | I | Lipid transport and metabolism | 38 | 286 | 4.1 |
| 0753 | Catalase | P | Inorganic ion transport and metabolism | 6 | 80 | 7.2 |
| 1613 | ABC-type sulfate transport system, periplasmic component | P | Inorganic ion transport and metabolism | 10 | 148 | 8.0 |
| 2223 | Nitrate/nitrite transporter | P | Inorganic ion transport and metabolism | 10 | 168 | 9.1 |
| 3158 | K+ transporter | P | Inorganic ion transport and metabolism | 115 | 394 | 1.9 |
| 3256 | Nitric oxide reductase large subunit | P | Inorganic ion transport and metabolism | 13 | 118 | 4.9 |
| 3696 | Putative silver efflux pump | P | Inorganic ion transport and metabolism | 254 | 938 | 2.0 |
| 4208 | ABC-type sulfate transport system, permease component | P | Inorganic ion transport and metabolism | 11 | 113 | 5.6 |
| 4651 | Kef-type K+ transport system, predicted NAD-binding component | P | Inorganic ion transport and metabolism | 29 | 171 | 3.2 |
| 4774 | Outer membrane receptor for monomeric catechols | P | Inorganic ion transport and metabolism | 40 | 365 | 5.0 |
| 1020 | Non-ribosomal peptide synthetase modules and related proteins | Q | Secondary metabolites biosynthesis, transport and catabolism | 92 | 895 | 5.3 |
| 3321 | Polyketide synthase modules and related proteins | Q | Secondary metabolites biosynthesis, transport and catabolism | 94 | 621 | 3.6 |
| **Information storage and processing** | | | | | | |
| 0751 | Glycyl-tRNA synthetase, beta subunit | J | Translation, ribosomal structure and biogenesis | 38 | 214 | 3.1 |
| 1187 | 16S rRNA uridine-516 pseudouridylate synthase and related pseudouridylate synthases | J | Translation, ribosomal structure and biogenesis | 74 | 348 | 2.6 |
| 0557 | Exoribonuclease R | K | Transcription | 71 | 336 | 2.6 |
| 0583 | Transcriptional regulator | K | Transcription | 182 | 888 | 2.7 |
| 3437 | Response regulator containing a CheY-like receiver domain and an HD-GYP domain | KT | Multiple classes | 30 | 213 | 3.9 |
| 0708 | Exonuclease III | L | Replication, recombination and repair | 45 | 219 | 2.6 |
| 1643 | HrpA-like helicases | L | Replication, recombination and repair | 86 | 315 | 2.0 |
| 4389 | Site-specific recombinase | L | Replication, recombination and repair | 7 | 112 | 8.7 |
| **Cellular processes and signaling** | | | | | | |
| 0729 | Outer membrane protein | M | Cell wall/membrane/envelope biogenesis | 16 | 124 | 4.2 |
| 1368 | Phosphoglycerol transferase and related proteins, alkaline phosphatase superfamily | M | Cell wall/membrane/envelope biogenesis | 9 | 126 | 7.6 |
| 1452 | Organic solvent tolerance protein OstA | M | Cell wall/membrane/envelope biogenesis | 29 | 178 | 3.3 |
| 4775 | Outer membrane protein/protective antigen OMA87 | M | Cell wall/membrane/envelope biogenesis | 81 | 337 | 2.3 |
| 1538 | Outer membrane protein | MU | Multiple classes | 106 | 624 | 3.2 |
| 1291 | Flagellar motor component | N | Cell motility | 10 | 128 | 7.0 |
| 0643 | Chemotaxis protein histidine kinase and related kinases | NT | Multiple classes | 64 | 348 | 3.0 |
| 0840 | Methyl-accepting chemotaxis protein | NT | Multiple classes | 43 | 442 | 5.6 |
| 1459 | Type II secretory pathway, component PulF | NU | Multiple classes | 96 | 370 | 2.1 |
| 2804 | Type II secretory pathway, ATPase PulE/Tfp pilus assembly pathway, ATPase PilB | NU | Multiple classes | 166 | 551 | 1.8 |
| 3419 | Tfp pilus assembly protein, tip-associated adhesin PilY1 | NU | Multiple classes | 11 | 113 | 5.6 |
| 5008 | Tfp pilus assembly protein, ATPase PilU | NU | Multiple classes | 21 | 256 | 6.6 |
| 0555 | ABC-type sulfate transport system, permease component | O | Posttranslational modification, protein turnover, chaperones | 5 | 78 | 8.5 |
| 0625 | Glutathione S-transferase | O | Posttranslational modification, protein turnover, chaperones | 92 | 352 | 2.1 |
| 3278 | Cbb3-type cytochrome oxidase, subunit 1 | O | Posttranslational modification, protein turnover, chaperones | 55 | 260 | 2.6 |
| 3484 | Predicted proteasome-type protease | O | Posttranslational modification, protein turnover, chaperones | 8 | 90 | 6.1 |
| 3634 | Alkyl hydroperoxide reductase, large subunit | O | Posttranslational modification, protein turnover, chaperones | 15 | 172 | 6.2 |
| 1391 | Glutamine synthetase adenylyltransferase | OT | Multiple classes | 46 | 238 | 2.8 |
| 0642 | Signal transduction histidine kinase | T | Signal transduction mechanisms | 343 | 962 | 1.5 |
| 0664 | cAMP-binding proteins - catabolite gene activator and regulatory subunit of cAMP-dependent protein kinases | T | Signal transduction mechanisms | 95 | 367 | 2.1 |
| 2199 | FOG: GGDEF domain | T | Signal transduction mechanisms | 121 | 1331 | 6.0 |
| 2200 | FOG: EAL domain | T | Signal transduction mechanisms | 88 | 1387 | 8.6 |
| 2202 | FOG: PAS/PAC domain | T | Signal transduction mechanisms | 49 | 381 | 4.2 |
| 2204 | Response regulator containing CheY-like receiver, AAA-type ATPase, and DNA-binding domains | T | Signal transduction mechanisms | 317 | 909 | 1.6 |
| 2206 | HD-GYP domain | T | Signal transduction mechanisms | 15 | 167 | 6.0 |
| 3434 | Predicted signal transduction protein containing EAL and modified HD-GYP domains | T | Signal transduction mechanisms | 2 | 70 | 19.0 |
| 3706 | Response regulator containing a CheY-like receiver domain and a GGDEF domain | T | Signal transduction mechanisms | 49 | 395 | 4.4 |
| 5000 | Signal transduction histidine kinase involved in nitrogen fixation and metabolism regulation | T | Signal transduction mechanisms | 42 | 234 | 3.0 |
| 5001 | Predicted signal transduction protein containing a membrane domain, an EAL and a GGDEF domain | T | Signal transduction mechanisms | 17 | 168 | 5.4 |
| 3267 | Type II secretory pathway, component ExeA (predicted ATPase) | U | Intracellular trafficking, secretion, and vesicular transport | 10 | 103 | 5.6 |
| 3451 | Type IV secretory pathway, VirB4 components | U | Intracellular trafficking, secretion, and vesicular transport | 7 | 99 | 7.7 |
| 4796 | Type II secretory pathway, component HofQ | U | Intracellular trafficking, secretion, and vesicular transport | 17 | 145 | 4.6 |
| 0841 | Cation/multidrug efflux pump | V | Defense mechanisms | 512 | 1693 | 1.8 |
| 1566 | Multidrug resistance efflux pump | V | Defense mechanisms | 104 | 376 | 2.0 |
| **Poorly characterized** | | | | | | |
| 2194 | Predicted membrane-associated, metal-dependent hydrolase | R | General function prediction only | 22 | 274 | 6.8 |
| 2509 | Uncharacterized FAD-dependent dehydrogenases | R | General function prediction only | 27 | 152 | 3.1 |
| 3178 | Predicted phosphotransferase related to Ser/Thr protein kinases | R | General function prediction only | 15 | 128 | 4.6 |
| 4783 | Putative Zn-dependent protease, contains TPR repeats | R | General function prediction only | 97 | 350 | 2.0 |
| 1795 | Uncharacterized conserved protein | S | Function unknown | 12 | 101 | 4.6 |
| 2354 | Uncharacterized protein conserved in bacteria | S | Function unknown | 2 | 72 | 19.5 |
| 3164 | Predicted membrane protein | S | Function unknown | 24 | 177 | 4.0 |
| 3220 | Uncharacterized protein conserved in bacteria | S | Function unknown | 10 | 103 | 5.6 |
| 5316 | Uncharacterized conserved protein | S | Function unknown | 5 | 82 | 8.9 |
| 5501 | Predicted secreted protein | S | Function unknown | 2 | 70 | 19.0 |

^a^The copy number of putative gene sequences in the CT and MC metagenomes and odds ratios (OR) between them are provided. The list of underrepresented COG groups is provided in Table S5.
