# Supplementary material for: Metagenomic Identification of Bacterioplankton Taxa and Pathways Involved in Microcystin Degradation in Lake Erie
Source: PLoS One. 2013 Apr 24;8(4):e61890. doi: 10.1371/journal.pone.0061890 (PMC3634838; doi:10.1371/journal.pone.0061890)
Supplement: Table S4 — Number and percent of metagenomic sequences with taxonomic assignment at different resolution levels. (DOC) [file pone.0061890.s005.doc]

Table S4. Number and percent of metagenomic sequences with taxonomic assignment at different resolution levels.

| **Parameter** | **CT-1** | **CT-2** | **MC-1** | **MC-2** |
| --- | --- | --- | --- | --- |
| Number (%) of total rRNA genes | 381 (0.2%) | 359 (0.2%) | 1000 (0.4%) | 771 (0.4%) |
| with order level annotation | 76 (20%) | 64 (18%) | 247 (25%) | 164 (21%) |
| with family level annotation | 54 (14%) | 40 (11%) | 243 (24%) | 154 (18%) |
| with genus level annotation | 54 (14%) | 40 (11%) | 223 (22%) | 139 (18%) |
| with species level annotation | 5 (1%) | 7 (2%) | 40 (4%) | 45 (6%) |
| Number ( %) of COG sequences with order level annotation | 27,266 (51%) | 30,736 (50%) | 96,623 (79%) | 73,393 (79%) |
| with family level annotation | 23,167 (43%) | 26,194 (42%) | 91,850 (75%) | 69,962 (75%) |
| with genus level annotation | 20,664 (38%) | 23,417 (38%) | 75,756 (62%) | 58,173 (63%) |
| with species level annotation | 19,440 (37%) | 22,071 (36%) | 70,431 (58%) | 54,194 (58%) |
