# Supplementary material for: Metagenomic Identification of Bacterioplankton Taxa and Pathways Involved in Microcystin Degradation in Lake Erie
Source: PLoS One. 2013 Apr 24;8(4):e61890. doi: 10.1371/journal.pone.0061890 (PMC3634838; doi:10.1371/journal.pone.0061890)
Supplement: Table S5 — List of underrepresented COG groups in the MC metagenomes relative to the CT metagenomes. (DOC) [file pone.0061890.s006.doc]

Table S5. List of underrepresented COG groups in the MC metagenomes relative to the CT metagenomes^a^.

| **COG** | **COG description** | **Class** | **Class description** | **CT** | **MC** | **OR_MC/CT_** |
| --- | --- | --- | --- | --- | --- | --- |
| **Metabolism** | | | | | | |
| 0554 | Glycerol kinase | C | Energy production and conversion | 93 | 44 | 0.25 |
| 0567 | 2-oxoglutarate dehydrogenase complex, dehydrogenase (E1) component, and related enzymes | C | Energy production and conversion | 223 | 175 | 0.42 |
| 0578 | Glycerol-3-phosphate dehydrogenase | C | Energy production and conversion | 56 | 25 | 0.24 |
| 1005 | NADH:ubiquinone oxidoreductase subunit 1 (chain H) | C | Energy production and conversion | 129 | 131 | 0.54 |
| 1007 | NADH:ubiquinone oxidoreductase subunit 2 (chain N) | C | Energy production and conversion | 123 | 130 | 0.57 |
| 1012 | NAD-dependent aldehyde dehydrogenases | C | Energy production and conversion | 584 | 671 | 0.62 |
| 1048 | Aconitase A | C | Energy production and conversion | 179 | 137 | 0.41 |
| 1071 | Pyruvate/2-oxoglutarate dehydrogenase complex, dehydrogenase (E1) component, eukaryotic type, alpha subunit | C | Energy production and conversion | 91 | 75 | 0.44 |
| 1085 | Galactose-1-phosphate uridylyltransferase | C | Energy production and conversion | 43 | 1 | 0.01 |
| 1139 | Uncharacterized conserved protein containing a ferredoxin-like domain | C | Energy production and conversion | 29 | 6 | 0.11 |
| 1304 | L-lactate dehydrogenase (FMN-dependent) and related alpha-hydroxy acid dehydrogenases | C | Energy production and conversion | 131 | 139 | 0.57 |
| 1529 | Aerobic-type carbon monoxide dehydrogenase, large subunit CoxL/CutL homologs | C | Energy production and conversion | 250 | 298 | 0.64 |
| 1804 | Predicted acyl-CoA transferases/carnitine dehydratase | C | Energy production and conversion | 242 | 305 | 0.67 |
| 1866 | Phosphoenolpyruvate carboxykinase (ATP) | C | Energy production and conversion | 38 | 19 | 0.26 |
| 2141 | Coenzyme F420-dependent N5,N10-methylene tetrahydromethanopterin reductase and related flavin-dependent oxidoreductases | C | Energy production and conversion | 143 | 91 | 0.34 |
| 2838 | Monomeric isocitrate dehydrogenase | C | Energy production and conversion | 45 | 27 | 0.32 |
| 3288 | NAD/NADP transhydrogenase alpha subunit | C | Energy production and conversion | 86 | 81 | 0.50 |
| 0006 | Xaa-Pro aminopeptidase | E | Amino acid transport and metabolism | 143 | 153 | 0.57 |
| 0076 | Glutamate decarboxylase and related PLP-dependent proteins | E | Amino acid transport and metabolism | 33 | 10 | 0.16 |
| 0119 | Isopropylmalate/homocitrate/citramalate synthases | E | Amino acid transport and metabolism | 202 | 194 | 0.51 |
| 0367 | Asparagine synthase (glutamine-hydrolyzing) | E | Amino acid transport and metabolism | 105 | 110 | 0.56 |
| 0404 | Glycine cleavage system T protein (aminomethyltransferase) | E | Amino acid transport and metabolism | 136 | 101 | 0.39 |
| 0520 | Selenocysteine lyase | E | Amino acid transport and metabolism | 185 | 119 | 0.34 |
| 0747 | ABC-type dipeptide transport system, periplasmic component | E | Amino acid transport and metabolism | 249 | 285 | 0.61 |
| 1176 | ABC-type spermidine/putrescine transport system, permease component I | E | Amino acid transport and metabolism | 61 | 40 | 0.35 |
| 1509 | Lysine 2,3-aminomutase | E | Amino acid transport and metabolism | 42 | 12 | 0.15 |
| 1748 | Saccharopine dehydrogenase and related proteins | E | Amino acid transport and metabolism | 39 | 21 | 0.28 |
| 2008 | Threonine aldolase | E | Amino acid transport and metabolism | 52 | 34 | 0.35 |
| 2303 | Choline dehydrogenase and related flavoproteins | E | Amino acid transport and metabolism | 172 | 182 | 0.56 |
| 2873 | O-acetylhomoserine sulfhydrylase | E | Amino acid transport and metabolism | 93 | 91 | 0.52 |
| 3200 | 3-deoxy-D-arabino-heptulosonate 7-phosphate (DAHP) synthase | E | Amino acid transport and metabolism | 50 | 33 | 0.35 |
| 3616 | Predicted amino acid aldolase or racemase | E | Amino acid transport and metabolism | 47 | 20 | 0.22 |
| 4608 | ABC-type oligopeptide transport system, ATPase component | E | Amino acid transport and metabolism | 73 | 62 | 0.45 |
| 0329 | Dihydrodipicolinate synthase/N-acetylneuraminate lyase | EM | Amino acid transport and metabolism | 172 | 164 | 0.51 |
| 0444 | ABC-type dipeptide/oligopeptide/nickel transport system, ATPase component | EP | Amino acid transport and metabolism | 140 | 128 | 0.49 |
| 1063 | Threonine dehydrogenase and related Zn-dependent dehydrogenases | ER | Amino acid transport and metabolism | 66 | 55 | 0.44 |
| 1387 | Histidinol phosphatase and related hydrolases of the PHP family | ER | Amino acid transport and metabolism | 34 | 11 | 0.17 |
| 0207 | Thymidylate synthase | F | Nucleotide transport and metabolism | 139 | 141 | 0.54 |
| 0208 | Ribonucleotide reductase, beta subunit | F | Nucleotide transport and metabolism | 155 | 147 | 0.51 |
| 0209 | Ribonucleotide reductase, alpha subunit | F | Nucleotide transport and metabolism | 379 | 522 | 0.74 |
| 0274 | Deoxyribose-phosphate aldolase | F | Nucleotide transport and metabolism | 51 | 12 | 0.12 |
| 0756 | dUTPase | F | Nucleotide transport and metabolism | 110 | 105 | 0.51 |
| 1351 | Predicted alternative thymidylate synthase | F | Nucleotide transport and metabolism | 101 | 56 | 0.29 |
| 1435 | Thymidine kinase | F | Nucleotide transport and metabolism | 39 | 16 | 0.22 |
| 1957 | Inosine-uridine nucleoside N-ribohydrolase | F | Nucleotide transport and metabolism | 43 | 14 | 0.17 |
| 2759 | Formyltetrahydrofolate synthetase | F | Nucleotide transport and metabolism | 61 | 40 | 0.35 |
| 0153 | Galactokinase | G | Carbohydrate transport and metabolism | 49 | 9 | 0.09 |
| 0205 | 6-phosphofructokinase | G | Carbohydrate transport and metabolism | 80 | 32 | 0.21 |
| 0395 | ABC-type sugar transport system, permease component | G | Carbohydrate transport and metabolism | 73 | 65 | 0.47 |
| 1070 | Sugar (pentulose and hexulose) kinases | G | Carbohydrate transport and metabolism | 77 | 33 | 0.23 |
| 1082 | Sugar phosphate isomerases/epimerases | G | Carbohydrate transport and metabolism | 120 | 43 | 0.19 |
| 1129 | ABC-type sugar transport system, ATPase component | G | Carbohydrate transport and metabolism | 97 | 61 | 0.33 |
| 1363 | Cellulase M and related proteins | G | Carbohydrate transport and metabolism | 42 | 9 | 0.11 |
| 1482 | Phosphomannose isomerase | G | Carbohydrate transport and metabolism | 26 | 2 | 0.04 |
| 1879 | ABC-type sugar transport system, periplasmic component | G | Carbohydrate transport and metabolism | 47 | 23 | 0.26 |
| 1929 | Glycerate kinase | G | Carbohydrate transport and metabolism | 31 | 6 | 0.10 |
| 2017 | Galactose mutarotase and related enzymes | G | Carbohydrate transport and metabolism | 44 | 21 | 0.25 |
| 2115 | Xylose isomerase | G | Carbohydrate transport and metabolism | 59 | 20 | 0.18 |
| 2271 | Sugar phosphate permease | G | Carbohydrate transport and metabolism | 63 | 19 | 0.16 |
| 3250 | Beta-galactosidase/beta-glucuronidase | G | Carbohydrate transport and metabolism | 25 | 4 | 0.08 |
| 3836 | 2,4-dihydroxyhept-2-ene-1,7-dioic acid aldolase | G | Carbohydrate transport and metabolism | 61 | 27 | 0.23 |
| 0095 | Lipoate-protein ligase A | H | Coenzyme transport and metabolism | 18 | 0 | 0 |
| 0214 | Pyridoxine biosynthesis enzyme | H | Coenzyme transport and metabolism | 33 | 11 | 0.17 |
| 0302 | GTP cyclohydrolase I | H | Coenzyme transport and metabolism | 76 | 64 | 0.45 |
| 0447 | Dihydroxynaphthoic acid synthase | H | Coenzyme transport and metabolism | 58 | 23 | 0.21 |
| 0499 | S-adenosylhomocysteine hydrolase | H | Coenzyme transport and metabolism | 155 | 160 | 0.55 |
| 1165 | 2-succinyl-6-hydroxy-2,4-cyclohexadiene-1-carboxylate synthase | H | Coenzyme transport and metabolism | 50 | 6 | 0.06 |
| 5598 | Trimethylamine:corrinoid methyltransferase | H | Coenzyme transport and metabolism | 30 | 6 | 0.10 |
| 0111 | Phosphoglycerate dehydrogenase and related dehydrogenases | HE | Coenzyme transport and metabolism | 101 | 94 | 0.50 |
| 1060 | Thiamine biosynthesis enzyme ThiH and related uncharacterized enzymes | HR | Coenzyme transport and metabolism | 60 | 12 | 0.10 |
| 0657 | Esterase/lipase | I | Lipid transport and metabolism | 99 | 84 | 0.45 |
| 1260 | Myo-inositol-1-phosphate synthase | I | Lipid transport and metabolism | 20 | 4 | 0.10 |
| 2084 | 3-hydroxyisobutyrate dehydrogenase and related beta-hydroxyacid dehydrogenases | I | Lipid transport and metabolism | 152 | 124 | 0.43 |
| 1028 | Dehydrogenases with different specificities (related to short-chain alcohol dehydrogenases) | IQR | Lipid transport and metabolism | 509 | 699 | 0.73 |
| 0226 | ABC-type phosphate transport system, periplasmic component | P | Inorganic ion transport and metabolism | 112 | 115 | 0.55 |
| 0529 | Adenylylsulfate kinase and related kinases | P | Inorganic ion transport and metabolism | 79 | 59 | 0.40 |
| 0569 | K+ transport systems, NAD-binding component | P | Inorganic ion transport and metabolism | 54 | 41 | 0.40 |
| 0803 | ABC-type metal ion transport system, periplasmic component/surface adhesin | P | Inorganic ion transport and metabolism | 33 | 17 | 0.27 |
| 1108 | ABC-type Mn2+/Zn2+ transport systems, permease components | P | Inorganic ion transport and metabolism | 36 | 14 | 0.20 |
| 1117 | ABC-type phosphate transport system, ATPase component | P | Inorganic ion transport and metabolism | 125 | 117 | 0.50 |
| 1119 | ABC-type molybdenum transport system, ATPase component/photorepair protein PhrA | P | Inorganic ion transport and metabolism | 26 | 1 | 0.02 |
| 3119 | Arylsulfatase A and related enzymes | P | Inorganic ion transport and metabolism | 722 | 178 | 0.13 |
| 3376 | High-affinity nickel permease | P | Inorganic ion transport and metabolism | 16 | 0 | 0 |
| 3540 | Phosphodiesterase/alkaline phosphatase D | P | Inorganic ion transport and metabolism | 63 | 45 | 0.38 |
| 1233 | Phytoene dehydrogenase and related proteins | Q | Secondary metabolites biosynthesis, transport and catabolism | 154 | 94 | 0.32 |
| 2124 | Cytochrome P450 | Q | Secondary metabolites biosynthesis, transport and catabolism | 166 | 107 | 0.34 |
| 3458 | Acetyl esterase (deacetylase) | Q | Secondary metabolites biosynthesis, transport and catabolism | 24 | 1 | 0.02 |
| 3653 | N-acyl-D-aspartate/D-glutamate deacylase | Q | Secondary metabolites biosynthesis, transport and catabolism | 80 | 36 | 0.24 |
| 5310 | Homospermidine synthase | Q | Secondary metabolites biosynthesis, transport and catabolism | 63 | 11 | 0.09 |
| **Information storage and processing** | | | | | | |
| 0154 | Asp-tRNAAsn/Glu-tRNAGln amidotransferase A subunit and related amidases | J | Translation, ribosomal structure and biogenesis | 269 | 104 | 0.49 |
| 0349 | Ribonuclease D | J | Translation, ribosomal structure and biogenesis | 53 | 102 | 0.22 |
| 0423 | Glycyl-tRNA synthetase (class II) | J | Translation, ribosomal structure and biogenesis | 95 | 108 | 0.18 |
| 1185 | Polyribonucleotide nucleotidyltransferase (polynucleotide phosphorylase) | J | Translation, ribosomal structure and biogenesis | 215 | 176 | 0.56 |
| 2519 | tRNA(1-methyladenosine) methyltransferase and related methyltransferases | J | Translation, ribosomal structure and biogenesis | 31 | 88 | 0.41 |
| 0085 | DNA-directed RNA polymerase, beta subunit/140 kD subunit | K | Transcription | 421 | 321 | 0.48 |
| 0086 | DNA-directed RNA polymerase, beta' subunit/160 kD subunit | K | Transcription | 332 | 180 | 0.49 |
| 0568 | DNA-directed RNA polymerase, sigma subunit (sigma70/sigma32) | K | Transcription | 201 | 96 | 0.28 |
| 1316 | Transcriptional regulator | K | Transcription | 34 | 147 | 0.43 |
| 1475 | Predicted transcriptional regulators | K | Transcription | 113 | 25 | 0.25 |
| 0553 | Superfamily II DNA/RNA helicases, SNF2 family | KL | Multiple classes | 248 | 589 | 0.65 |
| 1061 | DNA or RNA helicases of superfamily II | KL | Multiple classes | 314 | 87 | 0.49 |
| 0258 | Exonuclease III | L | Replication, recombination and repair | 167 | 344 | 0.48 |
| 0270 | HrpA-like helicases | L | Replication, recombination and repair | 113 | 89 | 0.26 |
| 0305 | Site-specific recombinase | L | Replication, recombination and repair | 358 | 10 | 0.16 |
| 0358 | 5'-3' exonuclease (including N-terminal domain of PolI) | L | Replication, recombination and repair | 197 | 13 | 0.22 |
| 0417 | Site-specific DNA methylase | L | Replication, recombination and repair | 184 | 48 | 0.37 |
| 0468 | Replicative DNA helicase | L | Replication, recombination and repair | 183 | 17 | 0.24 |
| 0507 | DNA primase (bacterial type) | L | Replication, recombination and repair | 52 | 24 | 0.29 |
| 0587 | DNA polymerase elongation subunit (family B) | L | Replication, recombination and repair | 483 | 11 | 0.11 |
| 0629 | RecA/RadA recombinase | L | Replication, recombination and repair | 94 | 17 | 0.14 |
| 0749 | ATP-dependent exoDNAse (exonuclease V), alpha subunit - helicase superfamily I member | L | Replication, recombination and repair | 379 | 29 | 0.28 |
| 0863 | DNA polymerase III, alpha subunit | L | Replication, recombination and repair | 179 | 104 | 0.49 |
| 1193 | Single-stranded DNA-binding protein | L | Replication, recombination and repair | 32 | 102 | 0.22 |
| 1796 | DNA polymerase I - 3'-5' exonuclease and polymerase domains | L | Replication, recombination and repair | 31 | 108 | 0.18 |
| 1961 | DNA modification methylase | L | Replication, recombination and repair | 68 | 176 | 0.56 |
| 3464 | Mismatch repair ATPase (MutS family) | L | Replication, recombination and repair | 37 | 88 | 0.41 |
| 3598 | DNA polymerase IV (family X) | L | Replication, recombination and repair | 44 | 321 | 0.48 |
| 3747 | Site-specific recombinases, DNA invertase Pin homologs | L | Replication, recombination and repair | 52 | 180 | 0.49 |
| 4570 | Transposase and inactivated derivatives | L | Replication, recombination and repair | 64 | 96 | 0.28 |
| 4581 | RecA-family ATPase | L | Replication, recombination and repair | 54 | 147 | 0.43 |
| **Cellular processes and signaling** | | | | | | |
| 4118 | Antitoxin of toxin-antitoxin stability system | D | Cell cycle control, cell division, chromosome partitioning | 29 | 5 | 0.09 |
| 5184 | Alpha-tubulin suppressor and related RCC1 domain-containing proteins | DZ | Multiple classes | 134 | 66 | 0.26 |
| 0438 | Glycosyltransferase | M | Cell wall/membrane/envelope biogenesis | 420 | 432 | 0.55 |
| 0463 | Glycosyltransferases involved in cell wall biogenesis | M | Cell wall/membrane/envelope biogenesis | 244 | 255 | 0.56 |
| 0472 | UDP-N-acetylmuramyl pentapeptide phosphotransferase/UDP-N-acetylglucosamine-1-phosphate transferase | M | Cell wall/membrane/envelope biogenesis | 136 | 149 | 0.58 |
| 0562 | UDP-galactopyranose mutase | M | Cell wall/membrane/envelope biogenesis | 48 | 17 | 0.19 |
| 0677 | UDP-N-acetyl-D-mannosaminuronate dehydrogenase | M | Cell wall/membrane/envelope biogenesis | 70 | 60 | 0.46 |
| 2089 | Sialic acid synthase | M | Cell wall/membrane/envelope biogenesis | 58 | 45 | 0.41 |
| 3306 | Glycosyltransferase involved in LPS biosynthesis | M | Cell wall/membrane/envelope biogenesis | 46 | 17 | 0.19 |
| 5434 | Endopolygalacturonase | M | Cell wall/membrane/envelope biogenesis | 30 | 5 | 0.08 |
| 0451 | Nucleoside-diphosphate-sugar epimerases | M | Cell wall/membrane/envelope biogenesis | 430 | 475 | 0.59 |
| 4948 | L-alanine-DL-glutamate epimerase and related enzymes of enolase superfamily | M | Cell wall/membrane/envelope biogenesis | 184 | 90 | 0.26 |
| 0071 | Molecular chaperone (small heat shock protein) | O | Posttranslational modification, protein turnover, chaperones | 76 | 59 | 0.41 |
| 0396 | ABC-type transport system involved in Fe-S cluster assembly, ATPase component | O | Posttranslational modification, protein turnover, chaperones | 74 | 67 | 0.48 |
| 0459 | Chaperonin GroEL (HSP60 family) | O | Posttranslational modification, protein turnover, chaperones | 309 | 304 | 0.52 |
| 0542 | ATPases with chaperone activity, ATP-binding subunit | O | Posttranslational modification, protein turnover, chaperones | 460 | 631 | 0.73 |
| 0719 | ABC-type transport system involved in Fe-S cluster assembly, permease component | O | Posttranslational modification, protein turnover, chaperones | 153 | 117 | 0.41 |
| 1030 | Membrane-bound serine protease (ClpP class) | O | Posttranslational modification, protein turnover, chaperones | 20 | 2 | 0.05 |
| 1331 | Highly conserved protein containing a thioredoxin domain | O | Posttranslational modification, protein turnover, chaperones | 45 | 13 | 0.15 |
| 0740 | Protease subunit of ATP-dependent Clp proteases | OU | Multiple classes | 125 | 132 | 0.56 |
| 1716 | FOG: FHA domain | T | Signal transduction mechanisms | 58 | 43 | 0.39 |
| 1132 | ABC-type multidrug transport system, ATPase and permease components | V | Defense mechanisms | 285 | 361 | 0.68 |
| 1403 | Restriction endonuclease | V | Defense mechanisms | 92 | 46 | 0.26 |
| **Poorly characterized** | | | | | | |
| 0595 | Predicted hydrolase of the metallo-beta-lactamase superfamily | R | General function prediction only | 59 | 34 | 0.31 |
| 0673 | Predicted dehydrogenases and related proteins | R | General function prediction only | 419 | 206 | 0.26 |
| 0714 | MoxR-like ATPases | R | General function prediction only | 254 | 295 | 0.62 |
| 1033 | Predicted exporters of the RND superfamily | R | General function prediction only | 61 | 40 | 0.35 |
| 1078 | HD superfamily phosphohydrolases | R | General function prediction only | 26 | 5 | 0.10 |
| 1090 | Predicted nucleoside-diphosphate sugar epimerase | R | General function prediction only | 48 | 11 | 0.12 |
| 1100 | GTPase SAR1 and related small G proteins | R | General function prediction only | 42 | 21 | 0.26 |
| 1216 | Predicted glycosyltransferases | R | General function prediction only | 103 | 77 | 0.40 |
| 1253 | Hemolysins and related proteins containing CBS domains | R | General function prediction only | 119 | 118 | 0.53 |
| 1373 | Predicted ATPase (AAA+ superfamily) | R | General function prediction only | 58 | 27 | 0.25 |
| 1408 | Predicted phosphohydrolases | R | General function prediction only | 32 | 13 | 0.21 |
| 1524 | Uncharacterized proteins of the AP superfamily | R | General function prediction only | 33 | 12 | 0.19 |
| 1735 | Predicted metal-dependent hydrolase with the TIM-barrel fold | R | General function prediction only | 28 | 0 | 0 |
| 1783 | Phage terminase large subunit | R | General function prediction only | 74 | 25 | 0.18 |
| 2252 | Permeases | R | General function prediction only | 27 | 4 | 0.07 |
| 2319 | FOG: WD40 repeat | R | General function prediction only | 90 | 61 | 0.36 |
| 2409 | Predicted drug exporters of the RND superfamily | R | General function prediction only | 106 | 10 | 0.05 |
| 2936 | Predicted acyl esterases | R | General function prediction only | 57 | 26 | 0.24 |
| 3179 | Predicted chitinase | R | General function prediction only | 150 | 67 | 0.24 |
| 3378 | Predicted ATPase | R | General function prediction only | 58 | 45 | 0.41 |
| 3552 | Protein containing von Willebrand factor type A (vWA) domain | R | General function prediction only | 29 | 5 | 0.09 |
| 3618 | Predicted metal-dependent hydrolase of the TIM-barrel fold | R | General function prediction only | 63 | 39 | 0.33 |
| 3740 | Phage head maturation protease | R | General function prediction only | 95 | 44 | 0.242 |
| 3772 | Phage-related lysozyme (muraminidase) | R | General function prediction only | 133 | 80 | 0.32 |
| 3889 | Predicted solute binding protein | R | General function prediction only | 81 | 33 | 0.21 |
| 3968 | Uncharacterized protein related to glutamine synthetase | R | General function prediction only | 34 | 12 | 0.18 |
| 4122 | Predicted O-methyltransferase | R | General function prediction only | 38 | 12 | 0.16 |
| 4626 | Phage terminase-like protein, large subunit | R | General function prediction only | 299 | 111 | 0.19 |
| 4653 | Predicted phage phi-C31 gp36 major capsid-like protein | R | General function prediction only | 41 | 24 | 0.31 |
| 4821 | Uncharacterized protein containing SIS (Sugar ISomerase) phosphosugar binding domain | R | General function prediction only | 21 | 3 | 0.07 |
| 4889 | Predicted helicase | R | General function prediction only | 32 | 14 | 0.23 |
| 5271 | AAA ATPase containing von Willebrand factor type A (vWA) domain | R | General function prediction only | 42 | 26 | 0.33 |
| 5362 | Phage-related terminase | R | General function prediction only | 129 | 48 | 0.20 |
| 5518 | Bacteriophage capsid portal protein | R | General function prediction only | 26 | 2 | 0.04 |
| 5525 | Bacteriophage tail assembly protein | R | General function prediction only | 79 | 68 | 0.46 |
| 5614 | Bacteriophage head-tail adaptor | R | General function prediction only | 30 | 11 | 0.19 |
| 0515 | Serine/threonine protein kinase | R | General function prediction only | 314 | 317 | 0.54 |
| 0391 | Uncharacterized conserved protein | S | Function unknown | 19 | 2 | 0.05 |
| 1262 | Uncharacterized conserved protein | S | Function unknown | 151 | 134 | 0.47 |
| 1520 | FOG: WD40-like repeat | S | Function unknown | 109 | 80 | 0.39 |
| 1615 | Uncharacterized conserved protein | S | Function unknown | 75 | 5 | 0.03 |
| 1692 | Uncharacterized protein conserved in bacteria | S | Function unknown | 31 | 13 | 0.22 |
| 2120 | Uncharacterized proteins, LmbE homologs | S | Function unknown | 66 | 15 | 0.12 |
| 2815 | Uncharacterized protein conserved in bacteria | S | Function unknown | 23 | 6 | 0.14 |
| 3007 | Uncharacterized paraquat-inducible protein B | S | Function unknown | 36 | 4 | 0.05 |
| 3108 | Uncharacterized protein conserved in bacteria | S | Function unknown | 43 | 16 | 0.20 |
| 3544 | Uncharacterized protein conserved in bacteria | S | Function unknown | 26 | 9 | 0.18 |
| 4102 | Uncharacterized protein conserved in bacteria | S | Function unknown | 134 | 74 | 0.29 |
| 4299 | Uncharacterized conserved protein | S | Function unknown | 22 | 3 | 0.07 |
| 4695 | Phage-related protein | S | Function unknown | 155 | 122 | 0.42 |
| 5323 | Uncharacterized conserved protein | S | Function unknown | 44 | 21 | 0.25 |
| 5410 | Uncharacterized protein conserved in bacteria | S | Function unknown | 151 | 55 | 0.19 |
| 0391 | Uncharacterized conserved protein | S | Function unknown | 59 | 34 | 0.31 |

^a^The copy number of putative gene sequences in the CT and MC metagenomes and odds ratios (OR) between them are provided.
