# Supplementary material for: Metagenomic Identification of Bacterioplankton Taxa and Pathways Involved in Microcystin Degradation in Lake Erie
Source: PLoS One. 2013 Apr 24;8(4):e61890. doi: 10.1371/journal.pone.0061890 (PMC3634838; doi:10.1371/journal.pone.0061890)
Supplement: Table S6 — Relative abundance (% of total sequences) of bacterial taxa revealed by 16S rRNA gene sequences in each metagenomes. (DOC) [file pone.0061890.s007.doc]

Table S6. Relative abundance (% of total sequences) of bacterial taxa revealed by 16S rRNA gene sequences in each metagenomes.

| **Taxa^a^** | **CT1** | **CT2** | **MC1** | **MC2** |
| --- | --- | --- | --- | --- |
| **Actinobacteria** |  |  |  |  |
| acI | 11.5 | 8.3 |  |  |
| acIII | 3.8 |  |  |  |
| acIV | 23.1 | 16.7 |  |  |
| Luna3 |  | 8.3 |  |  |
| **Bacteroidetes** |  |  |  |  |
| bacVI |  | 16.7 | 3.0 | 7.7 |
| **Planctomycetes** | 7.7 | 8.3 | 3.0 |  |
| **Proteobacteria** |  |  |  |  |
| **Alphaproteobacteria** |  |  |  |  |
| alfI |  |  |  | 2.6 |
| alfIV |  |  |  | 2.6 |
| alfV | 7.7 | 8.3 | 10.4 | 2.6 |
| alfVI |  |  | 1.5 |  |
| alfVIII | 3.7 |  |  |  |
| **Betaproteobacteria** |  |  |  |  |
| betI | 7.4 |  | 6.0 | 15.4 |
| betII |  |  | 1.5 |  |
| betIV | 7.4 | 8.3 | 60.0 | 66.7 |
| Unclassified Betaproteobacteria |  | 8.3 | 6.0 |  |
| **Gammaproteobacteria** |  |  |  |  |
| GamIII | 3.8 |  |  |  |
| GamV |  |  | 3.0 | 2.6 |
| **Verrucomicrobia** |  |  |  |  |
| Verl | 3.9 | 16.7 |  |  |
| CL120-10 (*Opitutaceae*) |  |  | 6.0 |  |
| Unclassified Verrucomicrobia | 15.4 |  |  |  |

^a^Taxonomic assignment is based on the “nearest neighbor” in the pre-made freshwater bacterial 16S rRNA gene ARB tree [4].

References:

[4] Newton, RJ, Jones SE, Eiler A, McMahon KD, Bertilsson S. (2011) A guide to the natural history of freshwater lake bacteria. Microbiol Mol Biol Rev 75: 14-49.
